# Combined valve and myocardial revascularization on a beating heart in high risk patients

**DOI:** 10.1186/1749-8090-10-S1-A161

**Published:** 2015-12-16

**Authors:** Paolo Pepino, Germano Coronella, Piermario Oliviero, Salvatore Giordano, Raffaela Provenzano, Agostino La Marca, Antonio Contaldo, Francesco Petteruti, Mario Monaco

**Affiliations:** 1 Department of Cardiothoracic Surgery, Presidio Ospedaliero Pineta Grande, Castel Voturno, Caserta, Italy; 2 Department of Vascular Surgery, Presidio Ospedaliero Pineta Grande, Castel Voturno, Caserta, Italy

## Background/Introduction

The management of complex high risk patients (pts) is always a challenge for the cardiac surgeon.

## Aims/Objectives

This paper describe our experience using an alternative surgical option in the treatment of concomitant valve disease and myocardial revascularization (CABG) in high risk patient with Ejection Fraction (EF) below 30 %.

## Method

From July 2013 up to September 2014 four high risk patients with low EF below 30 %, concomitant mitral valve disease (3 pts), ventricular aneurism (1 pt), and severe coronary artery disease were operated using on pump beating heart technique. Al pts were first connected to the IABP through the femoral artery and then put on CPB machine with a double venous cannula in the superior and inferior vena cava, aortic cannula, ventricular vent through the right superior pulmonary vein and cannula for the retrograde perfusion in the coronary sinus. At first was completed the myocardial revascularization with beating heart on pump, using the LIMA on the LAD in all patients and a segment of the saphenous vein or radial artery as a T graft from the left internal mammary artery (LIMA) for the lateral or posterior wall. Then the aorta was cross-clamped, leaving the heart beating while the coronary were perfused through the retrograde perfusion and through the composite grafts. The procedure was completed harvesting a mitral ring in three cases and in one case with an aneurismectomy of the left ventricle.

## Results

All patients were discharged 8 to 11 day after the operation, with an improved EF: Pt 1 EF pre op: 28%; post op EF 35%; Pt 2 EF pre op 30%; post op EF 40%; Pt 3 EF pre op 38%; post op EF 45%; Pt 4 pre op EF 30%; post op EF 40%.

## Discussion/Conclusion

Even if the study involve just a little number of patients, the technique can improve the post op ejection fraction without changing the in hospital stay. It can be proposed for that patients with a low EF in which there is a disease associated with CAD or in patient that have had a previous CABG with an heart disease different than a CAD.

